# Association of *VEGF* Gene Polymorphisms with Diabetic Retinopathy: A Meta-Analysis

**DOI:** 10.1371/journal.pone.0084069

**Published:** 2013-12-20

**Authors:** Jian-Yang Gong, Ye-Huan Sun

**Affiliations:** 1 Department of Epidemiology and Biostatistics, School of Public Health, Anhui Medical University, Hefei, Anhui, China; 2 Department of Ophthalmology, The First Affiliated Hospital of Anhui Medical University, Hefei, Anhui, China; Central China Normal University, China

## Abstract

**Background:**

Studies on the association of vascular endothelial growth factor (VEGF) gene -460T/C and -2578C/A polymorphisms with diabetic retinopathy (DR) have reported conflicting results. The aim of the present study was to assess the association by using meta-analysis.

**Methods:**

A systematic search of electronic databases (PubMed, EMBASE, Elsevier Science Direct, ISI Web of Science, CBM, CNKI and VIP) was carried out until Sept 18, 2013. The pooled odds ratios (ORs) and their corresponding 95% confidence intervals (CIs) were used to assess the strength of the association.

**Results:**

Eleven studies (-460T/C: 6 studies including 932 cases and 722 controls; -2578C/A: 6 studies including 1,071 cases and 1,137 controls) were involved in this meta-analysis. Significant association was found for -460T/C polymorphism (C versus T: OR=1.48, 95%CI=1.07–2.05, *P*=0.02; TC+CC versus TT: OR=1.78, 95%CI=1.02–3.12, *P*=0.04; CC versus TT+TC: OR=1.76, 95%CI=1.10–2.81, *P*=0.02), but not for -2578C/A polymorphism (*P*>0.05). Similar results were found in the subgroup analysis.

**Conclusions:**

This meta-analysis demonstrates that DR is associated with *VEGF* gene -460T/C polymorphism, but not -2578C/A polymorphism. Further case-control studies based on larger sample size are still needed, especially for -2578C/A polymorphism.

## Introduction

Diabetic retinopathy (DR) is the most common vascular complication of type 1 and type 2 diabetes mellitus characterized by increased vascular permeability, hemostatic abnormalities, increased tissue ischemia and neoangiogenesis [1,2]. It is one of the leading risk factors and causes of blindness worldwide [3]. To date, the pathogenesis of DR remains largely unknown, and is believed to be multifactorial. There is increasing evidence implicating genetic factors in the susceptibility to DR [4]. It is important to identify the genetic susceptibility factors for DR, which could help us to clarify the pathogenesis of DR.

Vascular endothelial growth factor (VEGF), a 45 KDa homodimeric glycoprotein, is secreted from various types of cells within the eye [5]. It has been shown to be an important mediator of retinal ischemia associated intraocular neovascularization [6]. *VEGF* is markedly upregulated in the eyes of the patients with DR, and *VEGF* antagonists can reduce retinal vascular permeability and neovascularization, which suggest that *VEGF* play an important role in the pathogenesis of DR [7,8]. Thus, *VEGF* is considered a plausible biological candidate for DR. Human *VEGF* gene is located on chromosome 6p21.3, and is highly polymorphic [9]. Many investigations have focused on the association between *VEGF* gene -460T/C (rs833061) and -2578C/A (rs699947) polymorphisms and DR risk [10-21]. However, the results were not all consistent. The conflicting results across these investigations reflects limitation in these studies, such as small sample size and ethnic difference.

Meta-analysis is a means of increasing the effective sample size under investigation through the pooling of data from individual association studies, thus enhancing the statistical power of the analysis for the estimation of genetic effects [22]. The aim of the present study is to perform a comprehensive meta-analysis to evaluate the association between *VEGF* gene -460T/C and -2578C/A polymorphisms and DR risk.

## Materials and Methods

### Publication search and inclusion criteria

This meta-analysis was conducted and reported according to the Preferred Reporting Items for Systematic Reviews and Meta-Analysis (PRISMA) Statement, issued in 2009 (Checklist S1). A systematic literature search in PubMed, EMBASE, Elsevier Science Direct, ISI Web of Science, China Biology Medical Literature Database (CBM), China National Knowledge Infrastructure (CNKI) and Database of Chinese Scientific and Technical Periodicals (VIP) was carried out to identify studies involving the association between *VEGF* gene -460T/C and -2578C/A polymorphisms and DR risk (last search updated on Sept 18, 2013). No language restrictions were applied. The keywords used for the search were “vascular endothelial growth factor OR VEGF”, “diabetic OR diabetes”, “retinopathy” and “gene OR allele OR polymorphism OR variation OR mutation”. Additional studies were identified by a hand search of references of original studies and review articles on the association of *VEGF* gene polymorphisms with DR. Analyses were performed for all cases with any form of DR compared with all diabetic without retinopathy. Eligible studies were selected according to the following explicit inclusion criteria: (1) evaluated the association between *VEGF* gene -460T/C or -2578C/A polymorphism and DR risk; (2) were a case-control study based on unrelated individuals; (3) sufficient genotype data were presented to calculate odds ratios (ORs) and 95% confidence intervals (CIs). When there were multiple publications from the same population, only the largest study was included. When a study reported the results on different ethnicities, we treated them independently.

### Data extraction

Two reviewers independently extracted the data with the standard protocol. Potential disagreements were resolved by discussion. The following items were collected from each study: the ﬁrst author’s name, year of publication, source of publication, racial ancestry, polymorphisms, the number of cases and controls, the available genotype and allele frequency information, diagnostic method of DR, and duration of diabetes. If original genotype frequency data were unavailable in relevant articles, a request for additional data were sent to the corresponding author.

### Statistical analysis

Allele frequencies for genetic polymorphisms in each study were determined by allele counting method. Hardy-Weinberg equilibrium (HWE) was assessed in each study by the Chi-square test. ORs with 95%CIs were calculated to assess the strength of the association between *VEGF* gene -460T/C and -2578C/A polymorphisms and DR risk. The pooled OR was calculated for dominant model, recessive model and allele comparisons. The existence of heterogeneity between studies was ascertained by the Chi square-test based Q-statistic [23]. We also measured the effect of heterogeneity by another measure, *I* ^2^=100%×(Q-df)/Q [24]. The pooled OR was calculated by the random effects model [25]. Meta-regression was performed to detect the source of heterogeneity [26]. The key contributor of the study to between-study heterogeneity was assessed by HETRED analysis (analysis for reducing heterogeneity by omitting a study using the STATA module of HETRED when *I* ^2^≥50%) [27]. The potential publication bias was examined visually in the funnel plot of ln[OR] against its standard error (SE). The degree of asymmetry of funnel flot was tested using Egger’s linear regression test [28]. If there is asymmetry, the regression line will not run through the origin. The intercept a provides a measure of asymmetry, the larger its deviation from zero the more pronounced the asymmetry. Harbord’s test was also used to detect the publication bias [29]. *P*<0.05 was considered representative of statistically significant publication bias. We performed subgroup analysis by ethnicity. We also performed subgroup analysis by types of DR if individual data were available. Studies not in HWE were subjected to a sensitivity analysis. All statistical analyses were performed using the software Review Manager 4.2 (The Cochrane Collaboration, Oxford, UK) and Stata 10.0 (STATA Corporation, College Station, Texas, USA). Two-sided *P*<0.05 was considered statistically significant.

## Results

### Literature search and characteristics

**Figure 1:**
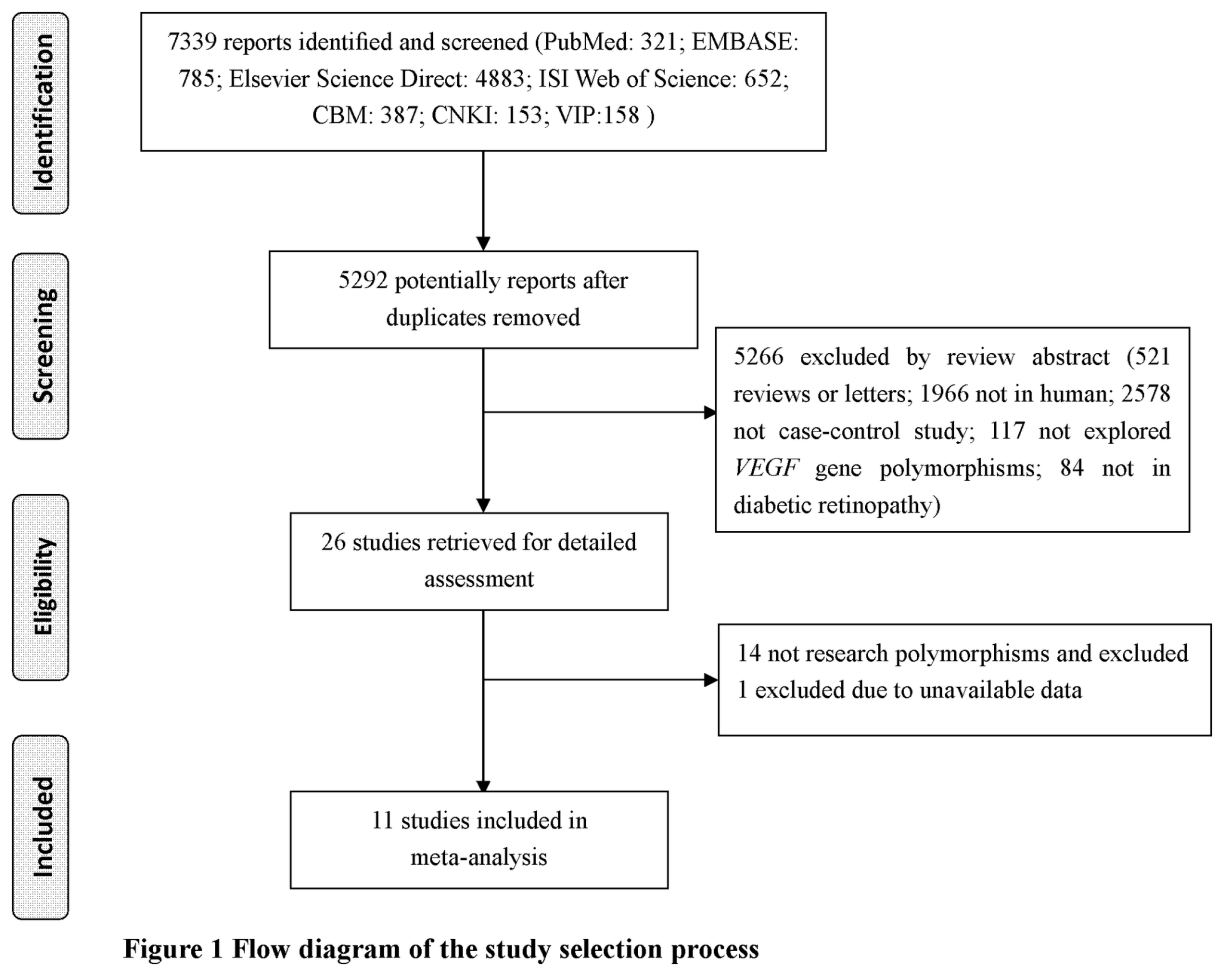
Flow diagram of the study selection process.

The selected study characteristics are summarized in Table 1 [10-15,17-21]. Figure 1 describes the flow of candidate and eligible papers. Initial search of the literature yielded 7,339 papers (PubMed: 321; EMBASE: 785; Elsevier Science Direct: 4,883; ISI Web of Science: 652; CBM: 387; CNKI: 153; VIP: 158). There were 5,292 potentially relevant papers after duplicates removed. We excluded 5,266 irrelevant papers on the basis of title and abstract (521 were review or letter; 1,966 were not conducted in human; 2,578 were not case-control study; 117 did not explore *VEGF* gene polymorphisms; 84 were not conducted in patients with DR). Twenty-six papers were retrieved and evaluated for compliance with the inclusion criteria. Of these papers, 15 were ineligible for the following reasons: 14 papers presented data on other polymorphisms, and 1 paper did not provide complete data [16]. Finally, a total of 11 studies were included in the meta-analysis (-460T/C: 6 studies including 932 cases and 722 controls; -2578C/A: 6 studies including 1,071 cases and 1,137 controls) [10-15,17-21]. In the eligible studies, there were 3 studies of Caucasian and 8 studies of Asian. The distributions of the genotypes in 3 studies were not in HWE [13,17,19].

**Table 1:** Characteristics of individual studies included in the meta-analysis^*^.

Polymorphism	Study	Year	Ethnic group	Type of diabetes	Diagnostic method of DR	Sample size (TT/TC/CC or CC/CA/AA)		Duration of diabetes	HWE		Result OR(95%CI)
						DR	DWR		DR	DWR	
-460T/C	Paine et al. [10]	2012	Asian	2	Fluorescein angiography Biomicroscopy	253 (152/81/20)	240 (167/67/6)	DR(15.0±8.0) DWR(17.0±5.0)	Yes	Yes	1.60(1.16-2.9)
	Yang et al. [11]	2011	Asian	2	Fundus photography Biomicroscopy	127 (65/46/16)	138 (81/52/5)	DR(14.6±7.5) DWR(15.1±4.4)	Yes	Yes	1.53(1.04-2.26)
	Szaflik et al. [17]	2008	Cauasian	2	Fluorescein angiography Ophthalmoscopy	153 (5/85/63)	61 (3/34/24)	NPDR(18.9±9.8) PDR(21.5±8.8) DWR(12.4±7.2)	No (0.0002)	No (0.039)	1.08(0.69-1.70)
	Wang et al. [18]	2008	Asian	2	Fundus photography Ophthalmoscopy	129 (19/73/37)	75 (26/33/16)	NPDR(13.6±3.6) PDR(14.9±2.8) DWR(13.2±3.2)	Yes	Yes	1.73(1.15-2.60)
	Suganthalakshmi et al. [19]	2006	Asian	2	Ophthalmoscopy Biomicroscopy	120 (36/81/3)	90 (61/29/0)	DR(15.5±6.9) DWR(17.0±8.0)	No (<0.0001)	Yes	2.96(1.84-4.77)
	Awata et al. [21]	2002	Asian	2	Fluorescein angiography Fundus photography	150 (79/58/13)	118 (52/57/9)	DR(12.9±7.9) DWR(7.3±6.8)	Yes	Yes	0.83(0.58-1.21)
-2578C/A	Yang et al. [11]	2011	Asian	2	Fundus photography Biomicroscopy	129 (66/47/16)	138 (82/51/5)	DR(14.6±7.5) DWR(15.1±4.4)	Yes	Yes	1.56(1.05-2.29)
	Chun et al. [12]	2010	Asian	2	Fundus photography Biomicroscopy Fluorescein angiography	253 (123/115/15)	134 (92/36/6)	DR(13.6±7.5) DWR(13.7±6.6)	Yes	Yes	1.84(1.28-2.66)
	Kangas-Kontio et al. [13]	2009	Cauasian	1,2	Fundus photography	126 (30/58/38)	96 (31/38/27)	DR(24.3±10.0) DWR(24.7±7.3)	Yes	No (0.043)	1.23(0.85-1.80)
	Abhary et al. [14]	2009	Cauasian	1,2	Fluorescein angiography Fundus photography	211 (48/109/54)	274 (71/134/69)	DR(T1DM:30.9±13.4; T2DM:17.5±8.6) DWR(T1DM:15.4±9.1; T2DM:12.9±8.7)	Yes	Yes	1.07(0.83-1.38)
	Nakamura et al. [15]	2009	Asian	2	Fundus photography Biomicroscopy	177 (85/70/22)	292 (163/107/22)	DR(22.7±8.9) DWR(16.7±7.5)	Yes	Yes	1.36(1.02-1.82)
	Awata et al. [20]	2005	Asian	2	Fundus photography Ophthalmoscopy Fluorescein angiography	175 (95/70/10)	203 (93/91/19)	DR(12.0±8.0) DWR(7.0±7.0)	Yes	Yes	0.74(0.54-1.02)

^*^ DR: diabetic retinopathy; DWR: diabetic without retinopathy; NPDR: non-proliferative diabetic retinopathy; PDR: proliferative diabetic retinopathy; T1DM: type 1 diabetes mellitus; T2DM: type 2 diabetes mellitus; HWE: Hardy–Weinberg equilibrium.

### Meta-analysis

**Figure 2:**
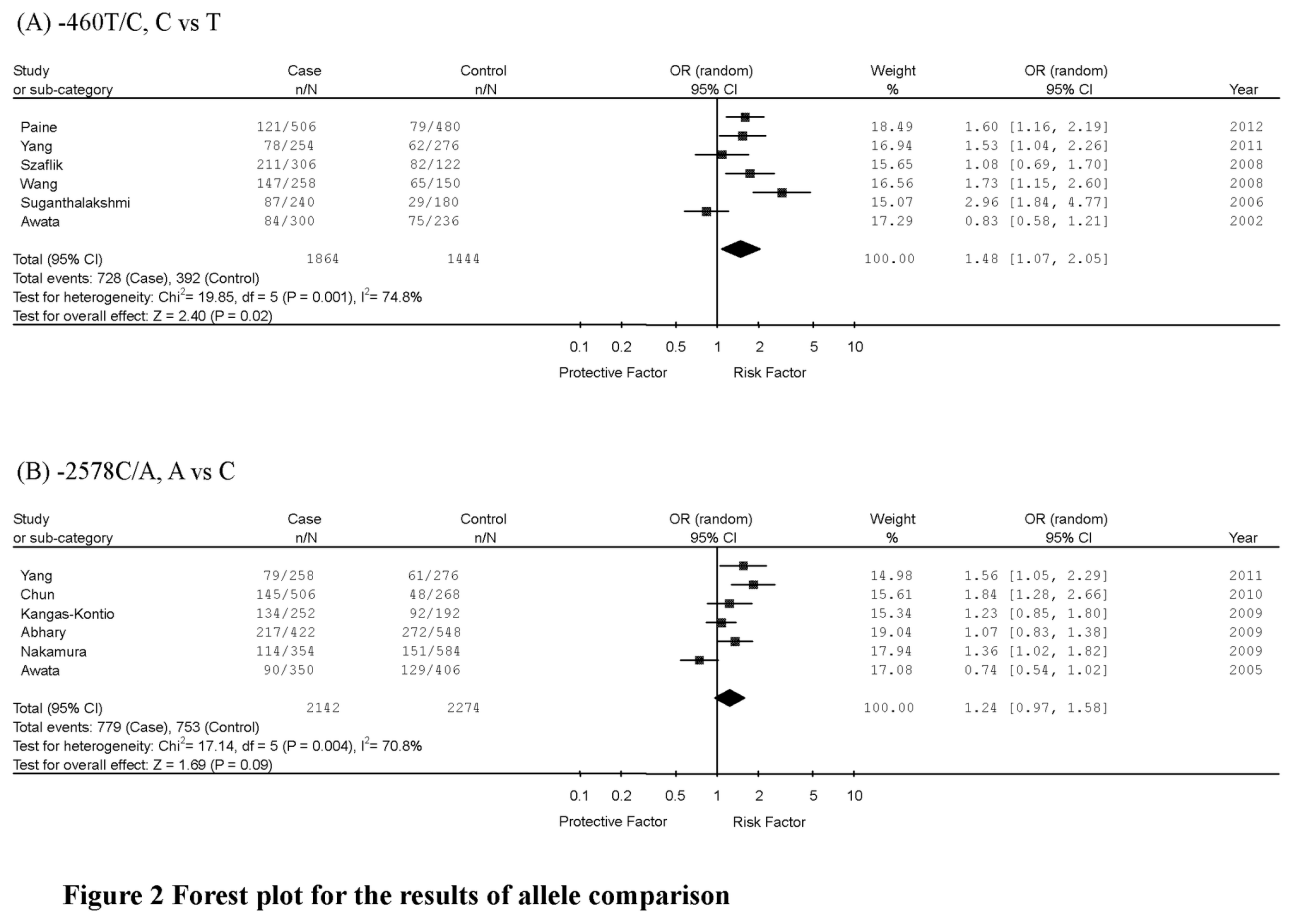
Forest plot for the results of allele comparison.

The summary of the meta-analysis for *VEGF* gene -460T/C and -2578C/A polymorphisms and DR risk is shown in Table 2 and Figure 2.

**Table 2:** Meta-analysis of *VEGF* gene polymorphisms and diabetic retinopathy association^*^.

Polymorphism	Comparsion		Sample size		No. of Studies	Test of association				Test of heterogeneity				Harbord's test (*P-value*)
			DR	DWR		*OR (95%CI)*	*Z*	*P-value*	Model	*χ^2^*		*P-value*	*I^2^(%)*	
-460T/C	Overall	C vs T	1864	1444	6	1.48(1.07-2.05)	2.40	0.02	R	19.85		0.001	74.8	0.862
		TC+CC vs TT	932	722	6	1.78(1.02-3.12)	2.01	0.04	R	28.80		<0.0001	82.6	0.637
		CC vs TT+TC	932	722	6	1.76(1.10-2.81)	2.37	0.02	R	8.04		0.15	37.8	0.212
	Asian	C vs T	1558	1322	5	1.57(1.09-2.27)	2.43	0.02	R	17.96		0.001	77.7	0.586
		TC+CC vs TT	779	661	5	1.81(0.99-3.32)	1.91	0.06	R	28.79		<0.0001	86.1	0.425
		CC vs TT+TC	779	661	5	2.07(1.24-3.45)	2.77	0.006	R	5.41		0.25	26.1	0.401
	PDR	C vs T	1178	1168	5	1.54(1.06-2.23)	2.29	0.02	R	14.18		0.007	71.8	0.763
		TC+CC vs TT	589	584	5	1.99(0.96-4.13)	1.86	0.06	R	23.41		0.0001	82.9	0.798
		CC vs TT+TC	589	584	5	1.65(1.10-2.47)	2.42	0.02	R	3.82		0.43	0.0	0.303
	NPDR	C vs T	432	508	3	1.10(0.69-1.76)	0.40	0.69	R	5.81		0.05	65.6	0.841
		TC+CC vs TT	216	254	3	1.33(0.47-3.73)	0.54	0.59	R	7.61		0.02	73.7	0.787
		CC vs TT+TC	216	254	3	1.10(0.69-1.76)	0.40	0.69	R	1.15		0.56	0.0	0.754
-2578C/A	Overall	A vs C	2142	2274	6	1.24(0.97-1.58)	1.69	0.09	R	17.14		0.004	70.8	0.442
		CA+AA vs CC	1071	1137	6	1.32(0.96-1.82)	1.71	0.09	R	15.67		0.008	68.1	0.686
		AA vs CC+CA	1071	1137	6	1.25(0.84-1.85)	1.10	0.27	R	9.89		0.08	49.5	0.453
	Asian	A vs C	1468	1534	4	1.29(0.88-1.91)	1.29	0.20	R	16.09		0.001	81.4	0.612
		CA+AA vs CC	734	767	4	1.33(0.82-2.14)	1,16	0.25	R	15.21		0.002	80.3	0.818
		AA vs CC+CA	734	767	4	1.46(0.72-2.96)	1.04	0.30	R	8.59		0.04	65.1	0.816
	Caucasian	A vs C	674	740	2	1.12(0.91-1.38)	1.07	0.28	R	0.36		0.55	0.0	−
		CA+AA vs CC	337	370	2	1.29(0.92-1.82)	1.46	0.14	R	0.46		0.50	0.0	−
		AA vs CC+CA	337	370	2	1.05(0.75-1.47)	0.27	0.78	R	0.04		0.83	0.0	−

^*^ *VEGF*: vascular endothelial growth factor; DR: diabetic retinopathy; DWR: diabetic without retinopathy; NPDR: non-proliferative diabetic retinopathy; PDR: proliferative diabetic retinopathy; vs: versus; R: random effects model.

#### Pooled effects for -460T/C polymorphism and DR risk

Six studies (932 cases and 722 controls) examining the association of *VEGF* gene -460T/C polymorphism with DR risk were included in the analysis. The C allele was considered as the risk variant. A significant association between -460T/C polymorphism and DR risk was identified (C versus T: OR=1.48, 95%CI=1.07-2.05, *P*=0.02, *P*_heterogeneity_=0.001; TC+CC versus TT: OR=1.78, 95%CI=1.02-3.12, *P*=0.04, *P*_heterogeneity_<0.0001; CC versus TT+TC: OR=1.76, 95%CI=1.10-2.81, *P*=0.02, *P*_heterogeneity_=0.15). To evaluate the race-specific effect, we conducted the subgroup analysis by ethnicity. Similar results were found in Asian population (C versus T: OR=1.57, 95%CI=1.09-2.27, *P*=0.02, *P*_heterogeneity_=0.001; TC+CC versus TT: OR=1.81, 95%CI=0.99-3.32, *P*=0.06, *P*_heterogeneity_<0.0001; CC versus TT+TC: OR=2.07, 95%CI=1.24-3.45, *P*=0.006, *P*_heterogeneity_=0.25). Meanwhile, we conducted the subgroup analysis by types of DR. Similar results were found in patients with proliferative diabetic retinopathy (C versus T: OR=1.54, 95%CI=1.06-2.23, *P*=0.02, *P*_heterogeneity_=0.007; TC+CC versus TT: OR=1.99, 95%CI=0.96-4.13, *P*=0.06, *P*_heterogeneity_=0.0001; CC versus TT+TC: OR=1.65, 95%CI=1.10-2.47, *P*=0.02, *P*_heterogeneity_=0.43).

#### Pooled effects for -2578C/A polymorphism and DR risk

Six studies (1,071 cases and 1,137 controls) investigated the association of *VEGF* gene -2578C/A polymorphism with DR risk and were included in the analysis. The A allele was considered as the risk variant. We found no association between -2578C/A polymorphism and DR risk (A versus C: OR=1.24, 95%CI=0.97-1.58, *P*=0.09, *P*_heterogeneity_=0.004; CA+AA versus CC: OR=1.32, 95%CI=0.96-1.82, *P*=0.09, *P*_heterogeneity_=0.008; AA versus CC+CA: OR=1.25, 95%CI=0.84-1.85, *P*=0.27, *P*_heterogeneity_=0.08). Similar results were found in Asian population (A versus C: OR=1.29, 95%CI=0.88-1.91, *P*=0.20, *P*_heterogeneity_=0.001; CA+AA versus CC: OR=1.33, 95%CI=0.82-2.14, *P*=0.25, *P*_heterogeneity_=0.002; AA versus CC+CA: OR=1.46, 95%CI=0.72-2.96, *P*=0.30, *P*_heterogeneity_=0.04) and Caucasian population (A versus C: OR=1.12, 95%CI=0.91-1.38, *P*=0.28, *P*_heterogeneity_=0.55; CA+AA versus CC: OR=1.29, 95%CI=0.92-1.82, *P*=0.14, *P*_heterogeneity_=0.50; AA versus CC+CA: OR=1.05, 95%CI=0.75-1.47, *P*=0.78, *P*_heterogeneity_=0.83).

### Evaluation of publication bias, heterogeneity and sensitivity

**Figure 3:**
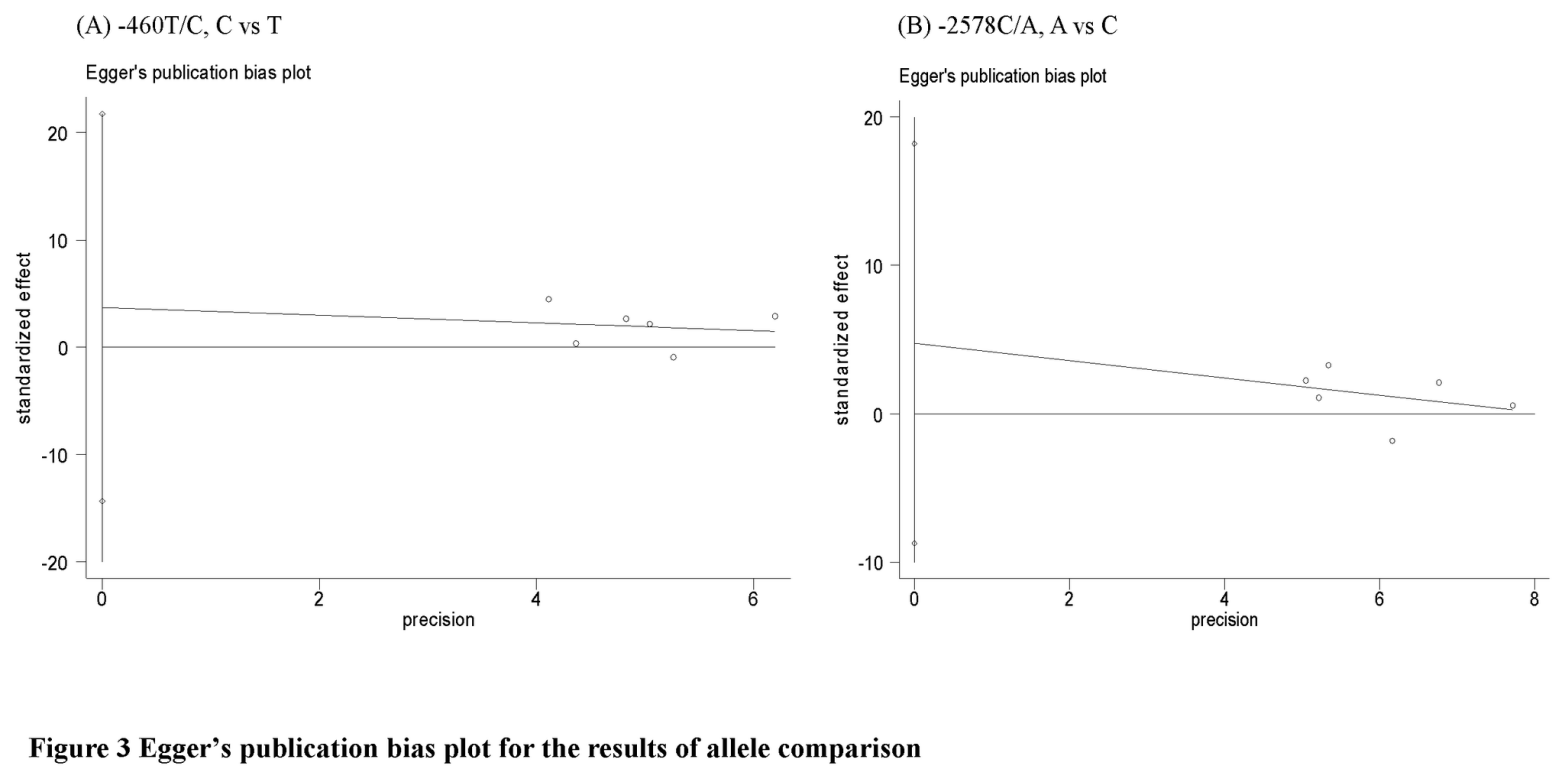
Egger’s publication bias plot for the results of allele comparison.

Publication bias was assayed by visual funnel plot inspection, Egger’s linear regression test (Table 3 and Figure 3) and Harbord’s test (Table 2). The funnel plots for -460T/C polymorphism (Overall population and Asian population) and -2578C/A polymorphism (Overall population and Asian population) were basically symmetric (funnel plots not shown), and Egger’s linear regression test and Harbord’s test did not indicate asymmetry of these plots (*P*>0.05). However, Egger’s linear regression test and Harbord’s test were not applied for -2578C/A polymorphism in Caucasian population due to the small number of studies. 

**Table 3:** Egger’s linear regression test to measure the funnel plot asymmetric^*^.

Polymorphism		Y axis intercept: *a* (*95%CI*)		
-460T/C		C vs T	TC+CC vs TT	CC vs TT+TC
	Overall	3.69(-14.37-21.75)	1.93(-6.95-10.80)	4.71(-2.03-11.46)
	Asian	6.92(-16.22-30.06)	6.28(-12.20-24.77)	4.44(-10.76-19.64)
	PDR	-0.32(-17.04-16.40)	1.18(-8.25-10.61)	3.85(-9.10-16.80)
	NPDR	6.12(-277.91-290.15)	1.82(-48.50-51.15)	-1.24(-39.86-37.38)
-2578C/A		A vs C	CA+AA vs CC	AA vs CC+CA
	Overall	4.74(-8.71-18.18)	3.38(-12.23-18.99)	1.63(-3.57-6.84)
	Asian	7.77(-39.51-55.04)	6.37(-53.33-66.08)	1.44(-20.25-23.12)

^*^ All *P*>0.05; NPDR: non-proliferative diabetic retinopathy; PDR: proliferative diabetic retinopathy; vs:versus.

As shown in Table 2, significant heterogeneity was found, thus meta-regression and HETRED analysis were conducted to detect the source of heterogeneity. For -460T/C polymorphism, meta-regression indicated that ethnicities (*P*=0.008) and types of DR (*P*=0.082) may contribute to heterogeneity. HETRED analysis indicated that the study by Suganthalakshmi et al. [19] and the study by Awata et al. [21] contribute to heterogeneity. Following exclusion of the two studies [19,21], association still existed (C versus T: OR=1.50, 95%CI=1.24-1.81, *I* ^2^=0.0%, *P*_heterogeneity_=0.44). For -2578C/A polymorphism, meta-regression indicated that ethnicities (*P*=0.064) and year of study (*P*=0.022) may contribute to heterogeneity. HETRED analysis indicated that the study by Abhary et al. [14] and the study by Awata et al. [20] contribute to heterogeneity. Following exclusion of the two studies [14,20], association was altered significantly (A versus C: OR=1.47, 95%CI=1.23-1.74, *I* ^2^=0.0%, *P*_heterogeneity_=0.45).

We conducted the sensitivity analysis excluding the studies not in HWE, which did not change the present results (-460T/C: C versus T: OR=1.37, 95%CI=1.15-1.65, *P*=0.0006, *P*_heterogeneity_=0.03; TC+CC versus TT: OR=1.41, 95%CI=0.85-2.34, *P*=0.19, *P*_heterogeneity_=0.005; CC versus TT+TC: OR=2.03, 95%CI=1.16-3.54, *P*=0.01, *P*_heterogeneity_=0.17; -2578C/A: A versus C: OR=1.24, 95%CI=0.92-1.66, *P*=0.15, *P*_heterogeneity_=0.002; CA+AA versus CC: OR=1.29, 95%CI=0.89-1.88, *P*=0.17, *P*_heterogeneity_=0.004; AA versus CC+CA: OR=1.31, 95%CI=0.79-2.16, *P*=0.30, *P*_heterogeneity_=0.04). The sensitivity analysis indicated that results of our study are stable and reliable.

## Discussion

Over the past three decades, the number of people with diabetes mellitus has more than doubled globally, making it one of the most important public health challenges to all nations [30]. DR occurs in 75% of patients with type 2 diabetes mellitus and almost all patients with type 1 diabetes mellitus within 15 years of the manifestation of diabetes [31,32]. Evidences support an important role for genetics in determining risk for DR [4]. Association studies are appropriate for searching susceptibility genes involved in DR, and, however, the results from these studies are usually conflicting and inconclusive. In this study, we systemically reviewed all available published studies and performed a meta-analysis to evaluate the association of *VEGF* gene -460T/C and -2578C/A polymorphisms with DR. Eleven studies (-460T/C: 6 studies including 932 cases and 722 controls; -2578C/A: 6 studies including 1,071 cases and 1,137 controls) were involved in this meta-analysis. Significant association was found for -460T/C polymorphism, but not for -2578C/A polymorphism. These results are strengthened by the facts that similar results were found in the subgroup analysis and that excluding the studies not in HWE gave us similar results. To our knowledge, the present meta-analysis is the first to assess the association between -460T/C polymorphism and DR. For -2578C/A polymorphism, in 2009, Abhary et al. [33] retrieved 2 studies [15,20] and performed a meta-analysis to evaluate the association of this polymorphism with DR. Their results revealed no significant association between -2578C/A polymorphism and DR, which are consistent with our results.

We detected a significant association between *VEGF* gene -460T/C polymorphism and DR risk. Recently, many growth factors have been implicated in having a role in the development of diabetic microvascular complications [34]. *VEGF*, one of the most common growth factors, can increase the permeability of the microvasculature, stimulate angiogenesis, and enhance collateral vessel formation [35]. Dysregulated *VEGF* expression is implicated in many disease pathologies, and *VEGF* levels have been found to be markedly elevated in the vitreous and aqueous fluids in the eyes of patients with DR [7]. *VEGF* may play an important role in the pathogenesis of DR. *VEGF* gene comprises a 14 kb-coding region with eight exons and seven introns [9]. Many polymorphisms have been described in the *VEGF* gene, and some polymorphisms influence levels of *VEGF* protein expression [36]. *VEGF* gene -460T/C polymorphism, one of the most frequently seen polymorphisms, is located at the -460 position in the promoter region. *VEGF* gene promoter region contains multiple regulatory elements, which involved in the regulation of *VEGF* gene expression, and -460T/C polymorphism C allele showed 70% increased promoter activity over T allele [36,37]. Therefore, we speculate that -460T/C polymorphism may increase expression of *VEGF*, resulting in a increased susceptibility to DR. Of course, the detailed mechanisms need further study. Additionally, the association may result from linkage disequilibrium with another functional polymorphism in the gene. Our results are further strengthened by the evidence from longitudinal data. In 2007, Al-Kateb et al. [38] utilized longitudinal data for retinal complications in the Caucasian type 1 diabetic population from the DCCT/Epidemiology of Diabetes Interventions and Complications (EDIC) Study to examine the role of *VEGF* gene polymorphisms, and their study provided strong evidence that more than one polymorphism in *VEGF* was independently associated with the risk of developing DR. In the current study, we did not detect a significant association between -2578C/A polymorphism and DR. However, evidence from HapMap database indicates that there is linkage disequilibrium between -460T/C and -2578C/A polymorphisms. Thus, further studies based on larger sample size are still needed to explore the association between -2578C/A polymorphism and DR.

Several specific details merit consideration in the current meta-analysis. First, only published studies were included in this meta-analysis. Meanwhile, Egger’s linear regression test and Harbord’s test were not applied for -2578C/A polymorphism in Caucasian population due to the small number of studies. Thus, publication bias may occur. A second consideration is signiﬁcant between-study heterogeneity was detected in some comparisons. Disease and population may contribute to the heterogeneity. For -2578C/A polymorphism, when excluding the two studies [14,20] that contribute to heterogeneity, association was altered significantly. Thus, signiﬁcant between-study heterogeneity may be distorting the meta-analysis. A third consideration is that analyses were not stratified by other factors such as age, gender and presence of nephropathy, and a more precise analysis stratified by other factors could be performed if individual data were available. Finally, this meta-analysis was limited by small sample size. For -2578C/A polymorphism, the statistical power of the subgroup analysis might not be sufficient to detect a significant association.

In conclusion, this meta-analysis demonstrates that DR is associated with *VEGF* gene -460T/C polymorphism, but not -2578C/A polymorphism. Due to the aforementioned limitations, further case-control studies based on larger sample size are still needed, especially for -2578C/A polymorphism.

## Supporting Information

Checklist S1 **Prisma checklist.**
(DOC)
